# Diagnosis and Treatment of Superficial Fungal Infections

**DOI:** 10.3390/jof11090669

**Published:** 2025-09-12

**Authors:** Suzana Otašević, Aleksandra Ignjatović

**Affiliations:** 1 Medical Faculty, University of Nis, 18000 Nis, Serbia; aleksandra.ignjatovic@medfak.ni.ac.rs; 2 Public Health Institute Nis, 18000 Nis, Serbia

On a global scale, one of the major challenges associated with superficial fungal infections (SFIs) of the skin and mucosa is their extremely high prevalence, along with the considerable proportion of patients who develop chronic infections [1].

The primary causative agents of skin and nail SFIs are dermatophytes (multicellular fungi), molds with a strong affinity for keratinized mammalian tissues. Superficial dermatophytoses are characterized by a slow but persistent progression of infection, in which virulence factors (adhesion factors and the production of keratinase and proteinases) contribute to tissue damage. The extent of this damage depends on the site of infection, the causative agent, and the immune status of the host [2].

From an epidemiological point of view, the most common species worldwide are *Microsporum* (*M.*) *canis*, *M. audouinii*, *Trichophyton* (*T.*) *interdigitale*, *T. rubrum*, *T. tonsurans*, and *T. verrucosum*. However, the presence of certain species of dermatophytes that cause SFIs, as well as the localization of these infections, varies according to the geographical, socio-economic, and ecological characteristics of a region, as well as the habits and customs of the local populations [3,4].

Epidemiological studies aimed at determining the distribution patterns of dermatophyte species and monitoring infected patients are of great importance. Considering that most cases of dermatophytoses are diagnosed and treated empirically, identifying the spectrum of fungi present in a specific area aids a dermatologist’s choice of therapy and its duration. Additionally, standard mycological diagnostics for the isolation and identification of causative agents have many disadvantages, including requiring expert knowledge and being time consuming. Therefore, there is a need to overcome these limitations by designing and implementing new molecular tests. Although presented as a revolutionary technique, the diagnostic performance of molecular automated tests that provide results in a few days still largely depends on the strains present in a particular region, as well as on the test spectrum of strains [5].

In this Special Issue, a study conducted by Almada et al. is presented, which explored the etiology and predisposing factors of SFIs in northeastern Argentina and determined the domination of dermatophytes fungi (46%) as causative agents of these infections. Anthropophilic species (*T. tonsurans*, *T. rubrum*, and *Epidermophyton flocossum*) emerged as the most common species (contribution 1).

In addition to its importance for the diagnosis and treatment of individual patients, mycological examination is also essential for guiding broader epidemiological measures. In this particular case, although anthropophilic species generally cause infections of milder intensity and respond well to treatment, human-to-human transmission can arise, potentially leading to an outbreak. Therefore, in routine practice, epidemiological measures to prevent the spread of disease should always be recommended based on mycological findings. This is especially relevant since researchers have specifically highlighted overcrowding as a variable associated with the identification of dermatophytosis.

Yeasts of the genera *Candida* and *Malassezia* are typically only colonizers, but in a significant percentage of people, they could be the cause of SFIs. Although many antifungals from the polyene, imidazole, or triazole groups are reported as being effective in the literature, there are no official recommendations or guidelines for their use, except for the treatment of onychomycosis, which always requires systemic therapy [6]. Fungal susceptibility differs in vivo and in vitro: satisfactory in vitro susceptibility does not guarantee successful treatment, whereas in vitro resistance indicates likely ineffectiveness in vivo. The results of the study conducted by Randjelovic et al. showed that the prevalence of superficial candidosis has been steadily increasing in recent years (contribution 2).

Moreover, the proportion of infections caused by non-albicans *Candida* species, which represent resistant strains, is rising. A cluster analysis of species distribution patterns and in vitro antifungal susceptibility revealed that one-third of *Candida* isolates exhibited low sensitivity to the most commonly used antifungals, highlighting the need for official treatment guidelines for these fungal infections and mandatory mycological analyses, including antifungal susceptibility testing, particularly in cases of chronic or recurrent disease.

On the other hand, some superficial fungal infections are uncommon but very serious, presenting numerous challenges in diagnosis, treatment, and potential complications. One such clinical entity is fungal keratitis (FK)—a rare but severe disease that can lead to evisceration or even irreversible blindness [7].

With the potential to invade the corneal epithelium via endocytosis, both *Candida* spp. as well as non-dermatophytic molds are causative agents of FK. Among molds, the fungal genera *Aspergillus* and *Fusarium* are predominant [8]. The results presented in contribution 3 describe a case series of patients with FK due to *Candida* spp. infection as the most prevalent; however, *Fusarium* spp., *Aspergillus* spp., *Paecilomyces* spp., *Alternaria* spp., *Acremonium* spp., *Stemphylium* spp., *Albifimbria* spp., *Scedosporium* spp., and *Curvularia* spp. were also isolated and identified as causative agents of this eye disease (contribution 3).

Fungal keratitis is often misdiagnosed as a bacterial infection; thus, sampling for fungal cultures is not part of routine practice. Additionally, the growth of the mold culture can be time consuming, which prolongs the procedure and delays treatment, contributing to a worse clinical outcome. In addition, there is no standardized protocol for the treatment of FK. None of the reported single treatment approaches have been shown to be the most effective or cost-efficient.

Since FK often presents with non-specific symptoms, and advanced diagnostic methods such as molecular testing, in vivo confocal microscopy, or tomographic imaging are not widely available, significant challenges in timely diagnosis and management still remain [9]. An ophthalmologist’s consideration of fungal infection is crucially important, along with an awareness of the established risk factors highlighted in published studies (contact with vegetable matter, the use of topical steroids, eye surgery, and wearing contact lenses) (contribution 3), as well as conducting a full microbiological assessment.

Furthermore, the introduction and implementation of official guidelines with clear recommendations on therapy, the choice of drug (amphotericin B, voriconazole, natamycin, or a combination of antimycotics), drug dosage and duration, and routes of the antifungal application (intracameral, intrastromal, and intravitreal) would significantly improve the management of FK [10,11].

Until the introduction and establishment of official guidelines with recommended therapy requirements, efforts are being made to develop new preparations. Within this Special Issue, a study by Rapti et al. aimed to investigate the sustained release of antifungals using a new AmB film for the treatment of FK. The study demonstrated that this new approach can yield better results, as well as enable a personalized approach to these infections. Besides proving the sustained delivery of dimeric AmB, which can be used for the therapy of FK and provides the favorable outcome of retaining the cornea, the approach presented a facile in vitro microfluidic model that can serve as a basis for the development and testing of other ophthalmic antimicrobial therapies (contribution 4).

As previously stated, mycological diagnostics should provide a rapid and accurate detection of potential causative agents within an optimal timeframe. The lack of implementation of a microbiological examination, including mycological testing, is often justified by the lengthy duration of conventional analyses. Therefore, the top priority in investigating fungal infections is the development and establishment of prompt, precise tests with high sensitivity and specificity, such as immunochromatographic ‘point-of-care’ assays and molecular techniques [12,13]. Molecular investigation enables a better understanding of the ecological, epidemiological, and taxonomic characteristics of fungi, primarily dermatophytes, and has contributed to an immeasurable improvement in the diagnosis of infections [5]. The possibility of the faster detection of a pathogen directly in patient samples/material significantly speeds up diagnostics, alongside the introduction of effective therapy, with a positive impact on the outcome of the infection. An investigation of the EUROArray Dermatomycosis Platform assay, which enables rapid and accurate fungal detection, revealed some disadvantages but indicated that this could be a valuable asset in clinical microbiology laboratories (contribution 5). A high diagnostic performance in terms of sensitivity (78.6%), specificity (91.8%), positive predictive value (75.9%), and negative predictive value (92.8%) make this test, with complementary methods, an excellent choice in routine clinical practice for enabling prompt and appropriate diagnosis, therapy, prescribing of appropriate epidemiological measures, and avoiding overtreatment in patients.

Additionally, to improve diagnostic procedures, the results reported by the Putek et al. study showed that dermoscopy and ultraviolet-enhanced fluorescence dermoscopy (UEFD) increase the accuracy of diagnosis and are helpful in assessing the effectiveness of *Kerion Celsi* treatment (contribution 6). *Kerion Celsi*, a serious scalp infection caused by zoophilic species of the genera *Microsporum* and *Trichophyton*, besides intense skin inflammation and the destruction of hair follicles, can influence scarring alopecia [2]. Essentially, timely diagnosis, as well as the initiation of appropriate treatment, can prevent adverse outcomes. The advantages of new molecular testing methods, which allow for the direct detection of the causative agent in a patient’s samples, are well known, but unfortunately, the methods are not readily available to most laboratories [14]. Consequently, applying dermoscopy and UEFD, which potentiate the detection of perifollicular celadon green fluorescence, represents a method that is a non-invasive, fast, easy-to-use diagnostic procedure that provides the diagnosis of fungal infections and differentiation from bacterial infections, psoriasis, alopecia areata, and scarring associated with systemic diseases.

In addition to dermatophytes, including yeasts of the genus *Candida* and *Malassezia*, SFIs caused by non-dermatophytic molds have been reported more frequently in recent years [2]. Non-dermatophytic molds are present and widespread in the human environment and can lead to human infection, including dermatomycosis. Skin infections can be primary; in these cases, the most common risk factor for non-dermatophyte mold infection is damage to intact skin, such as extensive burns [15]. Although many different genera of fungi inhabit the human environment, the most common causes of skin infections are species of the *Aspergillus flavus* and the *Aspergillus niger* complexes. It should also be emphasized that in immunocompromised patients, skin infected with these fungi can be a source of fungemia, resulting in the dissemination of pathogens and the development of invasive fungal infections.

In this Special Issue, Nacea et al. reported on the challenges in the diagnosis and treatment of skin aspergillosis in pediatric patients with burns. The authors excellently documented cases of primary cutaneous aspergilosis in teenagers with extensive burns, where the Meek micrografting technique with polyamide gauze that covers micrografts was applied. This procedure, although a good choice for treating burns, creates an ideal environment for fungal growth and the occurrence of mold infection on the skin, including aspergillosis. Moreover, the authors highlighted that the lack of a unique protocol for the treatment of cutaneous aspergillosis, especially in pediatric patients, makes the treatment (the choice of antimycotics, the application method, the most effective combination of systemic and topical antifungal drugs) of these patients a major challenge. However, in this case series study, the combined use of systemic and local applications of voriconazole was effective and associated with good outcomes of cutaneous aspergillosis in pediatric patients with extensive burns. Additionally, it was also emphasized that the monitoring of patients for the possible occurrence of mold infection and early diagnosis with laboratory-based evidence, including antifungal susceptibility testing, are crucial for a favorable outcome of the disease (contribution 7).

Based on the results published in this Special Issue, we highlight that the increasing prevalence and different clinical forms of fungal infections, the broader spectrum of fungi that are possible causes of SFIs, established fast and accurate non-culture methods, the possibilities of using a greater number of antifungal drugs with different mechanisms of action, as well as the registered resistance of fungi to antimycotics all indicate that the empirical approach to diagnosis and treatment has to be overcome. The harmonization of official attitudes aimed at the standardization of guidelines and recommendations regarding diagnostic procedures and therapy would significantly reduce the challenges of curing patients with SFIs.
